# Isolated Cerebral Mucormycosis Mimicking Meningioma in an Uncontrolled Diabetic Patient: A Case Report

**DOI:** 10.1002/ccr3.73361

**Published:** 2026-08-19

**Authors:** Sajid Khan, Kashif Qureshi, Bikramaditya Prasad Sah, V. Sureshkumar, Murtaja Satea, Bipin Chaurasia

**Affiliations:** ^1^ Department of Neurosurgery Prime Teaching Hospital, Peshawar Medical College Peshawar Pakistan; ^2^ Department of Neurosurgery Yale School of Medicine New Haven Connecticut USA; ^3^ Neurosurgery Department Madan Bhandari Academy of Health Sciences, Hetauda Hospital Hetauda Nepal; ^4^ Department of Neurosurgery Govt Mohan Kumaramangalam Medical College Hospital Salem Tamil Nadu India; ^5^ Department of Neurosurgery, College of Medicine University of Warith Al‐Anbiyaa Karbala Iraq; ^6^ Department of Neurosurgery Neurosurgery Clinic Birgunj Nepal

**Keywords:** case report, cerebral mucormycosis, diabetes mellitus, meningioma, mucormycosis

## Abstract

In a poorly controlled diabetic patient, isolated cerebral mucormycosis can mimic a meningioma on magnetic resonance imaging. The absence of a dural attachment at surgery was the first clue against meningioma, and histopathology was diagnostic. Early resection, antifungal therapy, and glycemic control were associated with a favorable outcome.

## 1 Introduction

Mucormycosis is an uncommon but rapidly progressive opportunistic infection caused by fungi of the order Mucorales. Its incidence differs markedly between regions, being infrequent in most high‐income countries and considerably higher in parts of South Asia [1]. The infection develops chiefly in immunocompromised hosts and in people with poorly controlled diabetes mellitus [1, 2]. Angioinvasion is its defining feature: fungal hyphae invade vessel walls, producing thrombosis, infarction and quickly advancing tissue necrosis [2]. Most infections follow inhalation or ingestion of sporangiospores, with traumatic inoculation being far less frequent [2].

Isolated cerebral mucormycosis (ICM), defined by disease confined to the brain without rhino‐orbital or paranasal sinus involvement, is a rare and distinct entity. It has traditionally been reported in people who inject drugs and in immunocompromised patients, and it carries a high case‐fatality rate [3, 4]. A systematic review of 55 immunocompetent adults with ICM found that 92% injected drugs and that more than 85% had lesions centered on the basal ganglia [4]. Although diabetes mellitus is the single most common metabolic risk factor for mucormycosis, and rhino‐cerebral disease is far more frequent among diabetic than among cancer patients [5], isolated cerebral involvement in a person with diabetes who does not inject drugs, presenting as a frontal‐lobe mass, remains distinctly unusual.

We describe such a patient, in whom ICM was mistaken for a meningioma, to underline the diagnostic difficulty, the decisive contribution of intraoperative and histopathological findings, and the principles of combined medical and surgical treatment. This case report has been reported in line with the SCARE 2025 criteria [6].

## 2 Case Presentation

### 2.1 History and Examination

A 28‐year‐old man with Type 2 diabetes mellitus, poorly controlled on irregular oral hypoglycemic agents, presented to the emergency department with a 2‐week history of progressive headache, blurring of vision and general malaise. He remained fully alert throughout, and the reported tiredness reflected constitutional malaise rather than any depression of consciousness. He had no preceding head trauma, no rhinological or orbital symptoms, and no previous neurosurgical admissions. He used only irregular oral hypoglycemic agents, with no regular corticosteroid or immunosuppressant therapy. He denied intravenous or other recreational drug use and reported no tobacco or alcohol use. Family history was noncontributory.

On arrival, his random blood glucose was 392 mg/dL, in keeping with poor glycemic control. He was conscious and fully oriented, with a Glasgow Coma Scale score of 15. Pupils were equal at 3 mm and reactive to light, and fundoscopy demonstrated bilateral papilledema. Tone, power (5/5 in all four limbs), sensation, and deep tendon reflexes were normal, with no cranial nerve deficit and no meningism. Blood pressure was 130/90 mmHg, heart rate 92 beats per minute, and respiratory rate 20 breaths per minute; he was afebrile. Cardiovascular and respiratory examinations were unremarkable.

### 2.2 Investigations

Blood cultures were sterile. Lumbar puncture yielded cerebrospinal fluid (CSF) with a white cell count of 13 cells/mm^3^, 11 red cells/mm^3^ and a protein of 45 mg/dL. The CSF glucose of 140 mg/dL, measured against a simultaneous serum glucose of 385 mg/dL, reflected the patient's systemic hyperglycemia rather than a true CSF‐to‐serum elevation. A potassium hydroxide (KOH) mount was not performed at presentation, because imaging had suggested a neoplasm and fungal infection was not initially considered. CSF cultures showed no growth. The laboratory findings are summarized in Table 1.

**TABLE 1:** Laboratory investigations at presentation.

Investigation	Result	Reference range	Interpretation
Random blood glucose	392 mg/dL	70–140 mg/dL	Marked hyperglycemia
CSF white cell count	13 cells/mm^3^	0–5 cells/mm^3^	Mild pleocytosis
CSF red cell count	11 cells/mm^3^	0 cells/mm^3^	Minor blood contamination
CSF protein	45 mg/dL	15–45 mg/dL	Upper limit of normal
CSF glucose	140 mg/dL (serum 385 mg/dL)	About two‐thirds of serum	Elevated, reflecting systemic hyperglycemia
Blood cultures	Sterile	No growth	No bacteremia
CSF cultures	No growth	No growth	No bacterial growth
KOH mount of CSF	Not performed	—	Fungal infection not initially suspected

*Note:* Reference ranges are approximate and laboratory‐dependent.

Abbreviations: CSF, cerebrospinal fluid; KOH, potassium hydroxide.

### 2.3 Imaging

**FIGURE 1:**
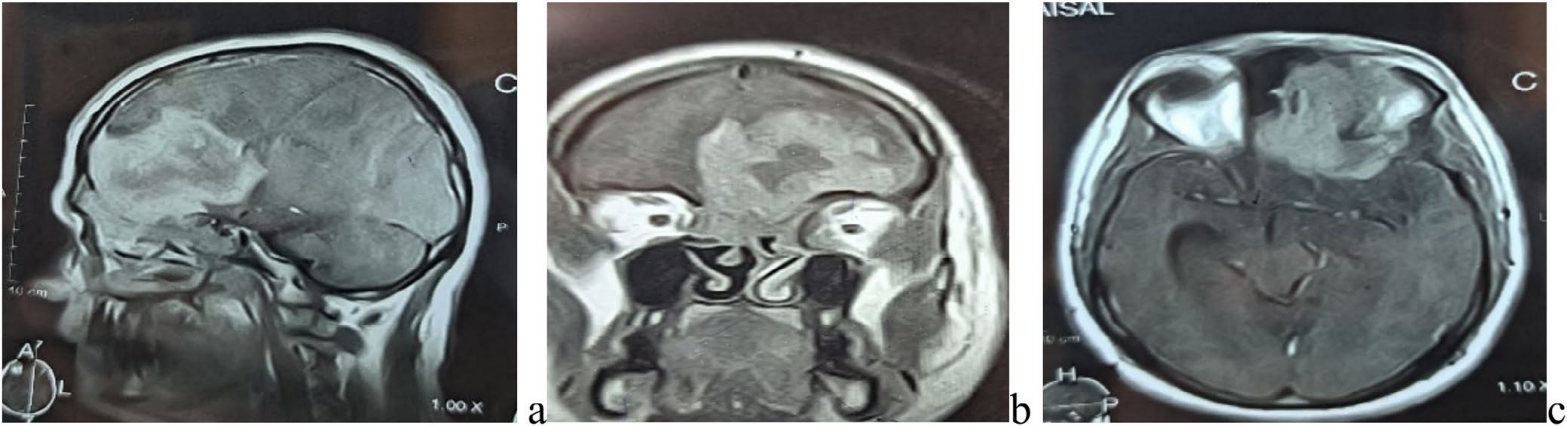
(a–c) Preoperative magnetic resonance imaging of the brain showing a well‐defined intra‐axial mass lesion in the left frontal lobe with surrounding vasogenic edema and mass effect, initially interpreted as a meningioma.

Magnetic resonance imaging of the brain (Figure 1) revealed a well‐defined intra‐axial mass in the left frontal lobe measuring approximately 4.2 × 3.8 × 3.5 cm. The lesion was isointense on T1‐weighted and hyperintense on T2‐weighted sequences, with surrounding vasogenic edema, mass effect, and heterogeneous enhancement after contrast. These appearances were initially read as consistent with a meningioma.

### 2.4 Differential Diagnosis

The radiological differential diagnosis for a solitary supratentorial mass in this patient included meningioma, high‐grade glioma, primary central nervous system lymphoma, solitary metastasis, cerebral abscess, and fungal granuloma. A dural‐based meningioma was favored at first because of the mass's well‐defined margins and avid, fairly homogeneous enhancement, features characteristic of meningioma [7], even though the apparently intra‐axial location was atypical for that diagnosis. High‐grade glioma remained plausible given the intra‐axial location and surrounding edema, although the sharp margins argued somewhat against it. Lymphoma was thought less likely without known immunosuppression and without the usual periventricular distribution and marked diffusion restriction. Metastasis was improbable in the absence of a known primary or of multiple lesions. A pyogenic abscess was not favored given the absence of fever, the modest CSF pleocytosis, and the solid rather than ring‐enhancing appearance. Fungal granuloma was not seriously entertained because pretest suspicion was low, and the correct diagnosis emerged only at operation and on histopathology.

### 2.5 Operation and Histopathology

**FIGURE 2:**
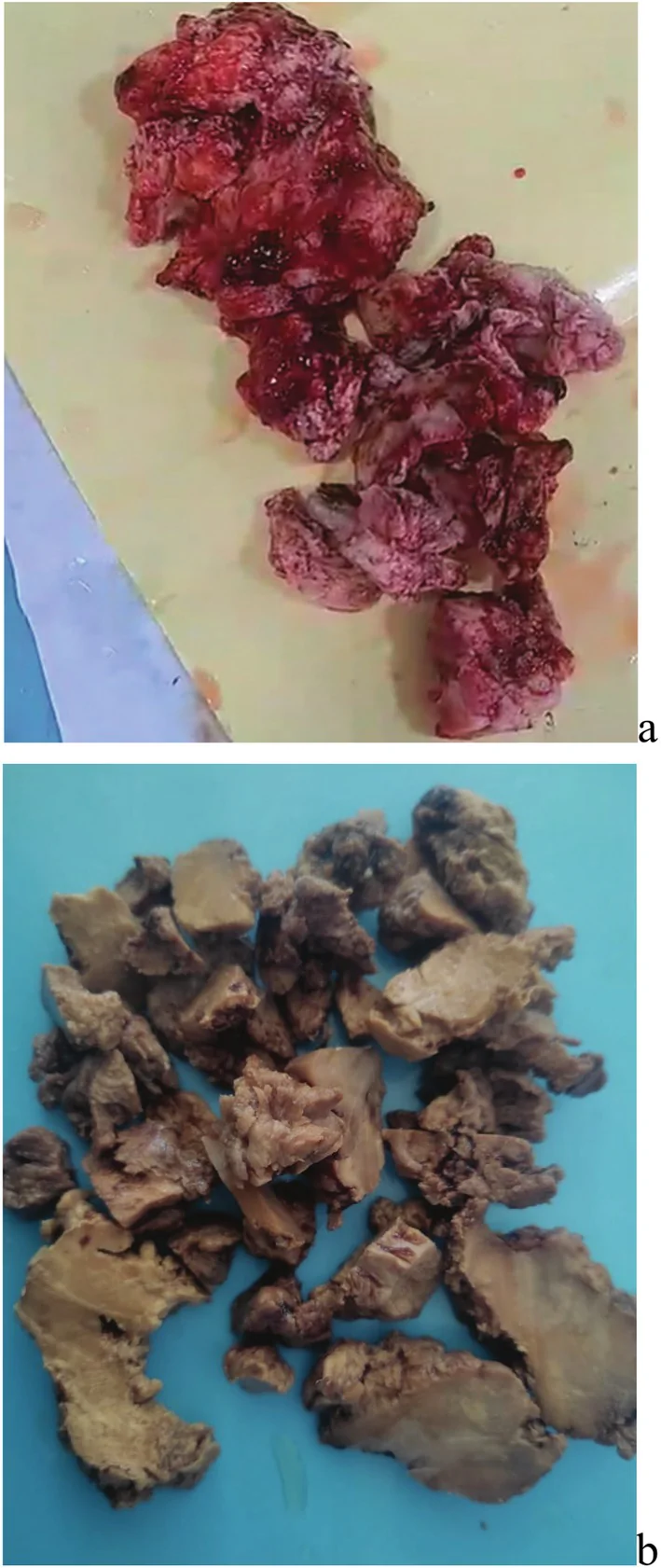
(a, b) Gross appearance of the excised intracranial lesion. (a) Resected tissue fragments at operation. (b) Further specimen fragments. The lesion was grayish‐white and friable.

Hyperglycemia was corrected preoperatively with an intravenous insulin infusion. The patient underwent a left frontal craniotomy by the attending neurosurgical team, planned as gross total resection of a presumed meningioma. Intraoperatively, the lesion was intra‐axial, grayish‐white, soft to friable, moderately vascular and irregular. No dural attachment was found, which argued against a typical meningioma; Simpson grading was therefore not applicable. The lesion was completely excised (Figure 2) and sent for histopathological examination.

**FIGURE 3:**
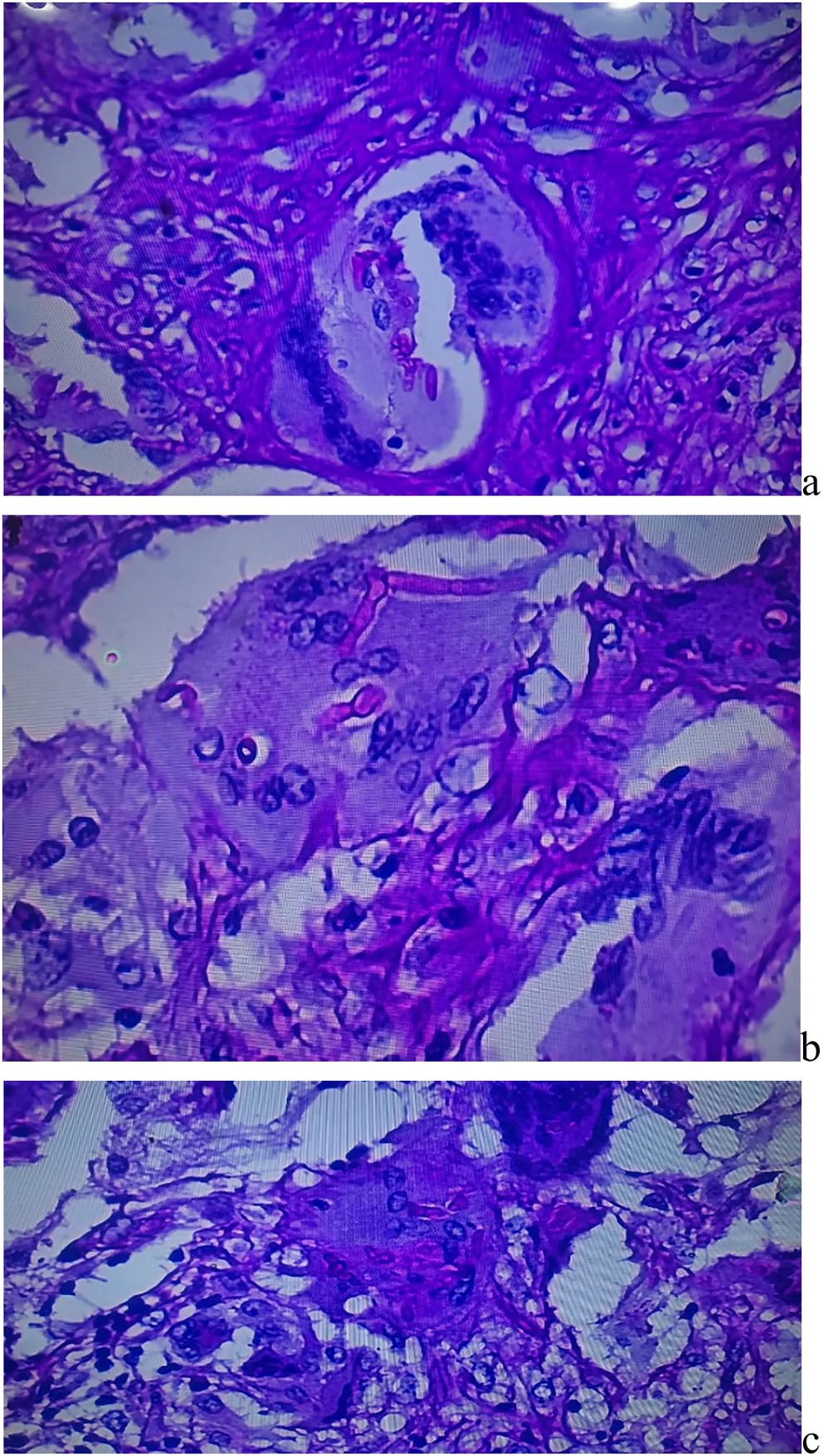
(a–c) Histopathology of the excised lesion, hematoxylin and eosin stain, showing broad, aseptate fungal hyphae with right‐angled branching invading the brain parenchyma, with associated necrosis and vascular invasion, consistent with cerebral mucormycosis. The three panels highlight the fungal hyphae and the surrounding necrosis and vascular invasion at successive fields.

Microscopy (Figure 3, hematoxylin and eosin) demonstrated broad, aseptate fungal hyphae with right‐angled branching invading the brain parenchyma, accompanied by extensive necrosis and vascular invasion, consistent with cerebral mucormycosis. The diagnosis rested on this characteristic hematoxylin and eosin morphology; special fungal stains, tissue culture, and molecular identification were not performed.

### 2.6 Outcome and Follow‐Up

Postoperatively, the patient received intravenous liposomal amphotericin B at 5 mg/kg/day for 4 weeks, followed by step‐down oral posaconazole delayed‐release tablets 300 mg once daily for a further 6 weeks. Empirical ceftriaxone 2 g twice daily and vancomycin 1 g twice daily were given for 7 days to cover possible bacterial co‐infection while the mild CSF pleocytosis was being investigated, and were stopped once cultures returned negative. Strict glycemic control was achieved with an intravenous insulin infusion, later converted to a subcutaneous regimen.

A postoperative computed tomography scan on Day 1 showed complete excision, resolution of mass effect, and no hemorrhage. Follow‐up MRI at 2 weeks confirmed no residual lesion. The patient improved steadily and was discharged on postoperative day 12 on continued antifungal and antidiabetic treatment. At 2 months he remained neurologically intact with no radiological recurrence, although this follow‐up interval is short for a disease capable of late relapse (see Limitations).

### 2.7 Timeline

The chronological sequence of presentation, investigation, treatment, and recovery is summarized in Table 2.

**TABLE 2:** Clinical timeline of presentation, investigation, treatment, and recovery.

Time point	Event
Two weeks before admission	Onset of progressive headache, blurred vision, and general malaise
Admission (day 0)	Random blood glucose 392 mg/dL; bilateral papilledema on examination; MRI and lumbar puncture performed
Day 0–1	Preoperative correction of hyperglycemia with intravenous insulin; left frontal craniotomy with gross total resection
Postoperative day 1	CT confirmed complete excision with no hemorrhage; histopathology reported cerebral mucormycosis
Postoperative weeks 1–4	Intravenous liposomal amphotericin B; empirical antibiotics for 7 days; strict glycemic control
Two weeks postoperatively	Follow‐up MRI showed no residual lesion
Postoperative day 12	Discharge on oral posaconazole and antidiabetic therapy
Postoperative weeks 5–10	Oral posaconazole step‐down (6 weeks total)
Two months	Neurologically intact, no radiological recurrence

## 3 Discussion

This patient illustrates the diagnostic and therapeutic challenges posed by isolated cerebral mucormycosis when it arises in poorly controlled diabetes. Cerebral involvement is among the most lethal presentations of the disease; a systematic review of rhino‐cerebral mucormycosis reported that diabetes carried the highest mortality of any predisposing condition, and untreated disease is almost invariably fatal [5, 8]. The mechanism is well described: hyperglycemia, ketoacidosis, and a rise in available free iron impair phagocyte function and favor fungal angioinvasion, which in turn drives rapid tissue necrosis [9].

Most reported cases of isolated cerebral mucormycosis have occurred in people who inject drugs, in whom the basal ganglia are characteristically involved [3, 4]. In the largest systematic review, 92% of immunocompetent patients with ICM injected drugs and more than 85% had basal ganglia disease [4]. Our patient departed from this pattern in two respects: he did not inject drugs, and the lesion lay in the frontal lobe. His case therefore reinforces poorly controlled diabetes as an independent risk factor for ICM and widens the recognized anatomical spectrum beyond the basal ganglia. Table 3 places the present case alongside previously reported intracranial mucormycosis mimicking tumors and highlights how it differs from both the drug‐use‐associated pattern and earlier diabetic cases.

**TABLE 3:** Reported cases of isolated or intracranial mucormycosis mimicking tumors, compared with the present case.

Study (year) [Ref.]	Population/predisposing factor	Lesion site	Imaging mimic	Distinction from present case
Meyerowitz et al. (2020) [4]	Immunocompetent, intravenous drug use (systematic review, *n* = 55)	Basal ganglia (> 85%)	Tumor‐like	No drug use and a frontal‐lobe lesion here
Verma et al. (2006) [3]	Isolated cerebral mucormycosis (case and review)	Basal ganglia predominant	Tumor‐like	Present case diabetic with a frontal‐lobe mass
Gupta et al. (2019) [10]	Immunocompetent infant	Intraventricular	Mass lesion	Different host and age; present case adult with poorly controlled diabetes
Al Barbarawi and Allouh (2015) [11]	Immunocompetent child	Intracranial	Mass lesion	Different host and age group
Uppal et al. (2023) [12]	Immunocompetent adult (fungal granuloma)	Intracranial	Meningioma	Similar meningioma mimic but non‐diabetic and granulomatous
Present case (2026)	Adult with poorly controlled Type 2 diabetes, no drug use	Left frontal lobe	Meningioma	Reference case

Mucormycosis can imitate neoplasia both radiologically and clinically across organ systems. Intraventricular disease has been described in an immunocompetent infant, and isolated intracranial disease in an immunocompetent child [10, 11]. Beyond the central nervous system, the infection has been mistaken for malignancy in pulmonary and disseminated forms [13, 14], and intracranial fungal disease has been misread as a meningioma and as a skull base malignancy [12, 15]. The same diagnostic trap has recently been reported in the chest, where pulmonary mucormycosis in a poorly controlled diabetic transplant recipient mimicked malignancy [16]; the shared thread across these cerebral and pulmonary cases is uncontrolled hyperglycemia driving angioinvasive fungal growth that is readily confused with tumor. In our patient, the imaging suggestion of meningioma prompted craniotomy.

The first intraoperative clue against meningioma was the absence of any dural attachment, since meningiomas are extra‐axial, dural‐based tumors. Histopathology remains the diagnostic gold standard, showing broad, aseptate hyphae with right‐angled branching alongside angioinvasion, thrombosis, and necrosis [3]. The imaging features reported for cerebral mucormycosis, including vascular occlusion, infarction, and extensive edema from angioinvasion [17, 18, 19], overlap with those of high‐grade glioma and cerebral abscess; in retrospect, the intra‐axial location should have tempered the initial preference for meningioma and raised glioma, abscess, or an atypical infective mass higher in the differential. Our patient, however, showed predominantly a mass with surrounding edema rather than the frank infarction often emphasized in the literature, which further blurred the distinction from a neoplasm.

Several points of diagnostic reasoning deserve emphasis. Lumbar puncture is not routinely undertaken for an apparently neoplastic mass and carries a real risk of herniation when there is significant mass effect; here it was performed, after imaging review, to help exclude an infective or inflammatory process given the atypical presentation, and its modest findings did not initially point to a fungal cause. Two avoidable pitfalls followed. A KOH mount of CSF was not obtained at presentation because the lesion was assumed to be a tumor, yet direct microscopy is rapid, inexpensive, and can suggest a fungal etiology early. In addition, fungal‐specific histochemical stains such as periodic acid‐Schiff and Grocott methenamine silver, together with tissue culture and molecular testing, raise diagnostic certainty and should be requested whenever fungal infection is plausible.

Optimal management combines prompt surgical debridement, antifungal therapy, and correction of the underlying metabolic derangement [20]. In a review of 929 localized and disseminated cases, survival reached 70% with combined surgery and antifungal therapy, against 61% for amphotericin B alone, 57% for surgery alone, and only 3% when untreated [5]. In cerebral disease specifically, surgical debridement and liposomal amphotericin B have each been independently associated with survival, and combined liposomal amphotericin B with posaconazole outperformed monotherapy [8]. Liposomal amphotericin B is the first‐line agent because of its potency and comparatively favorable safety profile, with posaconazole or isavuconazole reserved for step‐down or salvage therapy [20, 21]. When posaconazole is used, the delayed‐release tablet and intravenous formulations achieve more reliable exposure than the older suspension, and therapeutic drug monitoring targeting a trough of at least 1 mg/L is recommended [20, 22]. Guidelines advise continuing antifungal therapy until clinical and radiological resolution and durable reversal of the predisposing state, which usually takes several months [20]. In this patient, the six‐week course was guided by rapid clinical recovery, complete resection with radiological clearance, and restoration of glycemic control; nonetheless, a longer course with radiological surveillance would ordinarily be prudent, and the relatively short total duration here is a limitation. Early excision served the dual purpose of establishing the diagnosis and reducing fungal burden before antifungal step‐down and strict glycemic control.

Prognosis remains guarded, particularly with delayed diagnosis, extensive vascular involvement, or persistent hyperglycemia. Early recognition, timely surgery, aggressive antifungal therapy, and metabolic control are the main determinants of outcome.

## 4 Limitations

Several limitations should be acknowledged. As a single case, these observations cannot be generalized, and the contribution of any individual component of treatment to the outcome cannot be isolated. The diagnosis rested on characteristic hematoxylin and eosin morphology alone; special fungal stains, tissue culture, and molecular identification to species level were not performed, so the causative genus and species remain unconfirmed and a related mold cannot be formally excluded. A KOH mount was not obtained at presentation, and a systematic search for an occult source elsewhere, including dedicated chest imaging (high‐resolution computed tomography or chest radiography) and respiratory sampling, was not detailed, so a subclinical pulmonary or disseminated focus cannot be entirely excluded. Imaging was confined to the sequences available, no follow‐up image is presented, and follow‐up extended only to 2 months, which is short for a disease capable of late relapse; longer radiological surveillance would be needed to confirm durable cure. Finally, the total antifungal course was shorter than is often recommended, and the outcome might differ with more prolonged therapy.

## 5 Conclusion

Isolated cerebral mucormycosis can closely mimic a meningioma in a poorly controlled diabetic patient and should be considered in the differential diagnosis of an intracerebral mass in this setting, particularly when the lesion proves intra‐axial and lacks a dural attachment at surgery. In this patient the diagnosis rested on histopathology without confirmatory fungal stains, culture, or molecular testing, so it is best regarded as histologically characteristic rather than microbiologically confirmed. With that caveat, early diagnosis followed by combined surgical resection, antifungal therapy, and glycemic control was associated with a favorable short‐term outcome, and prompt recognition of the atypical intraoperative findings was pivotal.

## 6 Learning Points

- In a poorly controlled diabetic patient, isolated cerebral mucormycosis can mimic a meningioma on MRI, even as an intra‐axial frontal‐lobe mass outside the classic basal ganglia distribution.
- The absence of a dural attachment at surgery is an important early clue against meningioma and should prompt consideration of an infective or infiltrative process.
- Histopathology is central to diagnosis, but confirmatory fungal stains, culture, and molecular testing should be requested whenever fungal infection is possible.
- Favorable outcomes depend on early surgical resection, first‐line liposomal amphotericin B with posaconazole step‐down, and strict glycemic control, followed by sufficiently long antifungal therapy and radiological surveillance.

## Author Contributions

**Sajid Khan:** conceptualization, investigation, writing – original draft, methodology. **Bipin Chaurasia:** writing – review and editing, visualization, validation, supervision. **Murtaja Satea:** validation. **Bikramaditya Prasad Sah:** supervision. **Kashif Qureshi:** supervision. **V. Sureshkumar:** visualization.

## Funding

The authors have nothing to report.

## Disclosure

Provenance and Peer Review: Not commissioned, externally peer reviewed.

Guarantor: Bipin Chaurasia.

## Ethics Statement

Ethical approval was not required for this single case report in accordance with the authors' local institutional policy.

## Consent

Written informed consent was obtained from the patient for publication of this case report and the accompanying images. A copy of the consent form is available for review by the Editor‐in‐Chief on request.

## Conflicts of Interest

The authors declare no conflicts of interest.

## Data Availability

The data that support the findings of this study are available from the corresponding author upon reasonable request.
